# Supplementary material for: Functional connectomics of affective and psychotic pathology
Source: Proc Natl Acad Sci U S A. 2019 Apr 15;116(18):9050–9. doi: 10.1073/pnas.1820780116 (PMC6500110; doi:10.1073/pnas.1820780116)
Supplement: Supplementary File [file pnas.1820780116.sapp.pdf]

SUPPLEMENTAL: Functional connectomics of affective and psychotic pathology

Justin T. Baker[a,b*], Daniel G. Dillon[b,c], Lauren M. Patrick[d], Joshua L. Roffman[b,e,f], Roscoe O. Brady, Jr.[a,b,g], Diego A. Pizzagalli[b,c], Dost Öngür[a,b], Avram J. Holmes[c,d,h*]

[a]Schizophrenia and Bipolar Disorder Program, McLean Hospital, Belmont, MA 02478

[b]Department of Psychiatry, Harvard Medical School, Boston, MA 02114

[c]Center for Depression, Anxiety and Stress Research, McLean Hospital, Belmont, MA 02478

[d]Department of Psychology, Yale University, New Haven, CT 06520

[e]Athinoula A. Martinos Center for Biomedical Imaging, Massachusetts General Hospital Charlestown, MA 02129

[f]Department of Psychiatry, Massachusetts General Hospital, Boston, MA 02114

[g]Department of Psychiatry, Beth Israel Deaconess Medical Center, Boston, MA 02114

[h]Department of Psychiatry, Yale University, New Haven, CT 06520

*Correspondence and requests for materials should be addressed to A.J.H. (email: avram.holmes@yale.edu) or to J.T.B. (email: jtbaker@partners.org)

www.pnas.org/cgi/doi/10.1073/pnas.1820780116

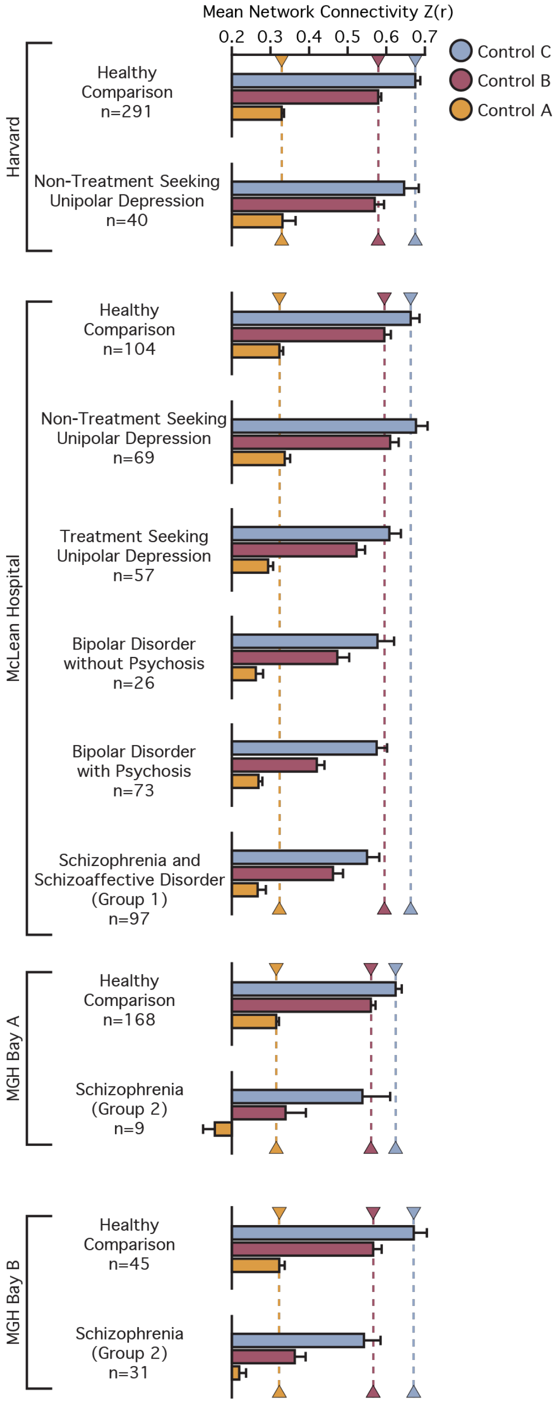

Supplemental Figure 1. Disruptions in frontoparietal network connectivity are evident across scanner and site of acquisition. Bar graphs reflect mean network connectivity for each group. Error bars denote standard error. Functional connectivity difference matrices were obtained by an analysis of variance of z-transformed Pearson correlation values, accounting for the effects of the coil, console software version, age, sex, race, ethnicity, and handedness.

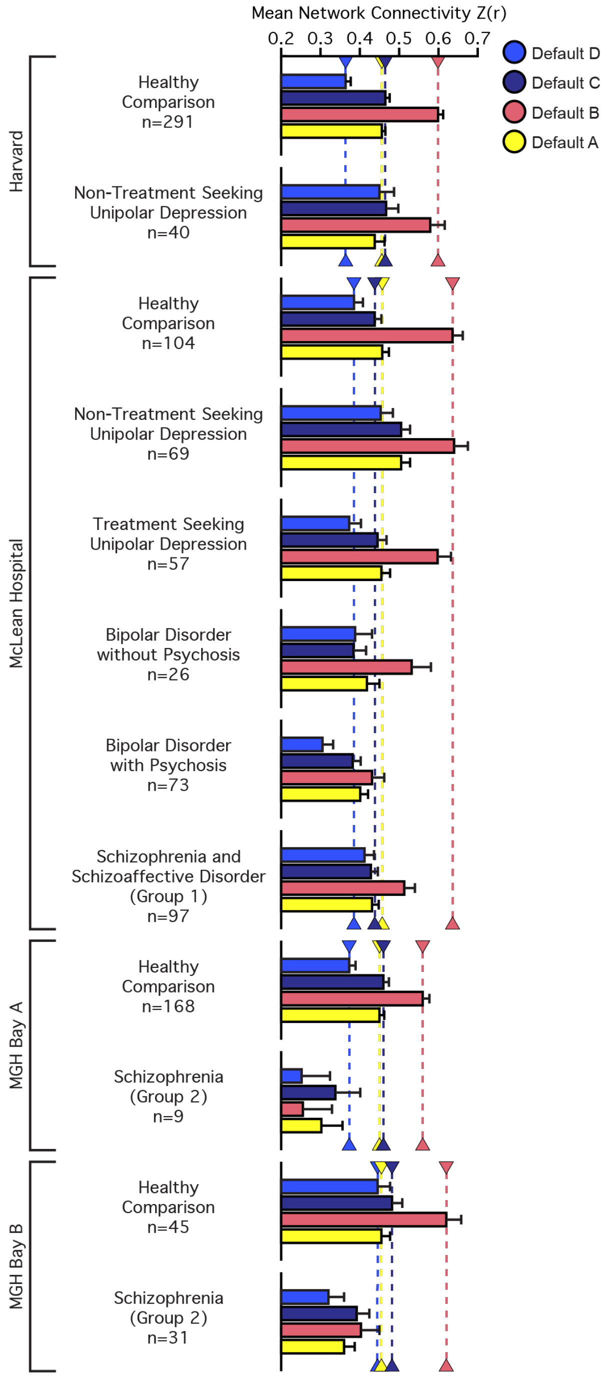

Supplemental Figure 2. Psychotic illness associates with a preferential reduction in default network integrity across scanner and site of acquisition. Bar graphs reflect mean network connectivity for each group within default A, B, C, and D. Error bars denote standard error. Functional connectivity difference matrices were obtained by an analysis of variance of z-transformed Pearson correlation values after linear regression of the effects of coil, console software version, age, sex, race, ethnicity, and handedness.

Supplemental Table 1. Participant demographics

	Healthy Comparison	Non-Treatment Seeking Depression	Treatment Seeking Depression	Bipolar Disorder (w/o psychosis)	Bipolar Disorder (psychosis)	Scz Group1	Scz Group 2			Healthy Comparison vs					
										NTSD	TSD	BD w/o	BD w/	SZ gp1	SZ gp2
Network	n=608	n=109	n=57	n=26	n=73	n=97	n=40	$F_{6,1003}$ /χ^2	p-value	p-values					
Age	33.6±13.5	30.1±10.9	35.0±13.7	38.7±15.3	31.3±11.4	33.3±12.0	44.8±10.6	1248.6	<0.001						****
Age range	18-71	18-64	18-68	20-71	18-63	18-65	20-62								
Percent female	44.9	66.1	57.9	53.8	35.6	32.0	22.5	41.5	<0.001	****				*	***
Percent right handed	91.4	100.0	89.5	92.3	84.9	84.5	80.0	30.3	<0.005	**				*	***
Percent White (not Hispanic or Latino)	65.1	65.2	80.4	69.2	75.3	70.1	67.5	4.5	0.62						
YMRS	N/A	N/A	n=54<br>4.46±3.96	n=24<br>5.71±3.78	n=73<br>21.70±11.15	n=92<br>14.26±8.89	N/A	49.88	<0.001	N/A	N/A	N/A	N/A	N/A	N/A
PANSS positive	N/A	N/A	n=53<br>8.89±2.38	n=24<br>9.08±2.23	n=73<br>19.33±7.44	n=92<br>18.77±6.53	n=37<br>18.11±5.99	39.46	<0.001	N/A	N/A	N/A	N/A	N/A	N/A
PANSS negative	N/A	N/A	14.53±4.66	12.38±3.45	10.64±4.49	15.89±8.03	21.49±5.12	22.44	<0.001	N/A	N/A	N/A	N/A	N/A	N/A
PANSS general	N/A	N/A	36.17±6.07	35.21±6.55	29.78±7.43	33.17±9.04	36.84±6.25	8.16	<0.001	N/A	N/A	N/A	N/A	N/A	N/A
Average CPZ Equivalent	N/A	N/A	N/A	N/A	n=73<br>380.65±277.69	n=92<br>408.22±361.55	N/A	0.29	0.59	N/A	N/A	N/A	N/A	N/A	N/A
Slice SNR	175.3±51.2	196.1±67.3	195.6±53.3	177.6±40.4	140.7±65.4	166.2±66.2	144.3±67.0	10.7	<0.001	**			****		*
Number of micro-movements	20.3±24.7	15.1±20.4	24.5±23.1	24.8±17.4	35.1±31.8	25.9±26.6	37.9±35.6	8.4	<0.001				****		****

*Note: NTSD: community non-treatment seeking depression; TSD: unipolar treatment seeking depression; BD w/o: bipolar disorder without psychosis; BD w/: bipolar disorder with psychosis; SZ gp1: schizophrenia group 1; SZ gp2: schizophrenia group 2. N/A: Not availableMicro-movements reflect the number of relative translations in 3D space ≥0.1 mm. *P*-values for group comparisons * ≤0.05 **≤0.01 *** ≤0.005 **** ≤0.001

Supplemental Table 1 Cont. Participant demographics

	Non-Treatment Seeking Depression vs					Treatment Seeking Depression vs				Bipolar Disorder (w/o psychosis) vs			Bipolar Disorder (psychosis) vs		Scz Group1 vs Scz Group2
	TSD	BD w/o	BD w/	SZ gp1	SZ gp2	BD w/o	BD w/	SZ gp1	SZ gp2	BD w/	SZ gp1	SZ gp2	SZ gp1	SZ gp2	
Network	p-values					p-values				p-values			p-values		p-values
Age			*		****				***					****	****
Age range															
Percent female			****	****	****		*	***	****		*	**			
Percent right handed	***	***	****	****	****										
Percent White (not Hispanic or Latino)															
YMRS	N/A	N/A	N/A	N/A	N/A		****	****	N/A	****	****	N/A	****	N/A	N/A
PANSS positive	N/A	N/A	N/A	N/A	N/A		****	****	****	****	****	****			
PANSS negative	N/A	N/A	N/A	N/A	N/A		****		****		**	****	****	****	****
PANSS general	N/A	N/A	N/A	N/A	N/A		****	*		***			***	****	*
Average CPZ Equivalent	N/A	N/A	N/A	N/A	N/A	N/A	N/A	N/A	N/A	N/A		N/A	N/A	N/A	N/A
Slice SNR			****	***	****		****	*	****	*			*		
Number of micro-movements			****	*	****										

*Note: NTSD: community non-treatment seeking depression; TSD: unipolar treatment seeking depression; BD w/o: bipolar disorder without psychosis; BD w/: bipolar disorder with psychosis; SZ gp1: schizophrenia group 1; SZ gp2: schizophrenia group 2. *P*-values for group comparisons * ≤0.05 **≤0.01 *** ≤0.005 **** ≤0.001

Supplemental Table 2. Network interactions affected by psychiatric illnesses with or without psychosis

	Healthy Comparison	Patients without psychosis	Patients with psychosis				HC vs Pw/o	HC vs PP	PP vs Pw/o
Network	n=608	n=192	n=210	$F_{2,1001}$	μ^2	p-value	p-values		
Default D	0.38±0.22	0.41±0.24	0.35±0.21	2.66	0.01	0.07	0.06	0.33	≤0.05
Default C	0.46±0.19	0.46±0.17	0.40±0.16	8.84	0.02	≤0.001	0.84	≤0.001	≤0.005
Default B	0.60±0.23	0.60±0.25	0.45±0.28	23.72	0.05	≤0.001	0.55	≤0.001	≤0.001
Default A	0.46±0.15	0.46±0.16	0.40±0.18	6.41	0.01	≤0.005	0.93	≤0.001	≤0.01
Control C	0.66±0.22	0.64±0.24	0.55±0.19	14.42	0.03	≤0.001	0.14	≤0.001	≤0.005
Control B	0.58±0.16	0.56±0.16	0.42±0.18	63.95	0.11	≤0.001	≤0.01	≤0.001	≤0.001
Control A	0.33±0.11	0.31±0.11	0.26±0.11	25.65	0.05	≤0.001	0.06	≤0.001	≤0.001
Limbic	0.38±0.26	0.33±0.28	0.31±0.27	6.52	0.01	≤0.005	0.08	≤0.001	0.21
Salience	0.45±0.13	0.45±0.13	0.38±0.14	20.39	0.04	≤0.001	0.18	≤0.001	≤0.001
Ventral attention	0.46±0.14	0.46±0.15	0.40±0.16	9.86	0.02	≤0.001	0.48	≤0.001	≤0.005
Dorsal attention B	0.48±0.17	0.50±0.17	0.45±0.19	3.01	0.01	≤0.05	0.35	≤0.05	≤0.05
Dorsal attention A	0.68±0.23	0.71±0.22	0.63±0.27	4.54	0.01	≤0.05	0.24	≤0.05	≤0.005
Somatomotor B	0.67±0.17	0.65±0.18	0.62±0.17	6.88	0.01	≤0.001	0.09	≤0.001	0.16
Somatomotor A	0.61±0.25	0.58±0.26	0.44±0.26	26.58	0.05	≤0.001	≤0.05	≤0.001	≤0.001
Visual peripheral	1.05±0.25	1.08±0.26	0.99±0.24	2.68	0.01	0.07	0.53	≤0.05	≤0.05
Visual central	0.72±0.31	0.80±0.32	0.84±0.27	14.83	0.03	≤0.001	≤0.01	≤0.001	0.08

*Note: Values reflect mean of raw z-transformed Pearson correlation values ± standard deviation across all regional interactions for that network; HC: healthy comparison participants; Pw/o: patients without psychosis; PP: patients with psychosis.

Supplemental Table 3. Network interactions affected by psychiatric illnesses with or without psychosis

	Healthy Comparison	Non-Treatment Seeking Depression	Treatment Seeking Depression	Bipolar Disorder (w/o psychosis)	Bipolar Disorder (psychosis)	Scz Group1	Scz Group 2				Healthy Comparison vs					
											NTSD	TSD	BD w/o	BD w/	SZ gp1	SZ gp2
Network	n=608	n=109	n=57	n=26	n=73	n=97	n=40	$F_{6,997}$	μ^2	p-value	p-values					
Default D	0.38±0.22	0.44±0.27	0.37±0.20	0.37±0.20	0.31±0.20	0.41±0.22	0.29±0.17	3.67	0.02	≤0.001	***			**		
Default C	0.46±0.19	0.48±0.18	0.45±0.15	0.38±0.14	0.38±0.14	0.42±0.17	0.36±0.14	4.98	0.03	≤0.001			*	****	*	*
Default B	0.60±0.23	0.63±0.25	0.59±0.25	0.49±0.22	0.44±0.26	0.51±0.28	0.35±0.30	9.47	0.05	≤0.001				****	****	****
Default A	0.46±0.15	0.48±0.16	0.46±0.14	0.40±0.16	0.41±0.17	0.43±0.19	0.34±0.16	3.45	0.02	≤0.005				**		****
Control C	0.66±0.22	0.68±0.25	0.60±0.22	0.55±0.21	0.57±0.20	0.55±0.19	0.54±0.17	5.79	0.03	≤0.001				****	****	*
Control B	0.58±0.16	0.61±0.16	0.51±0.14	0.44±0.16	0.42±0.18	0.46±0.18	0.35±0.16	25.20	0.13	≤0.001		***	****	****	****	****
Control A	0.33±0.11	0.34±0.11	0.29±0.10	0.25±0.08	0.28±0.11	0.28±0.11	0.20±0.08	12.71	0.07	≤0.001		*	****	****	****	****
Limbic	0.38±0.26	0.36±0.28	0.27±0.28	0.29±0.24	0.30±0.25	0.35±0.27	0.26±0.28	3.63	0.02	≤0.001		**		***		*
Salience	0.45±0.13	0.46±0.12	0.44±0.12	0.39±0.14	0.38±0.14	0.40±0.14	0.34±0.15	7.52	0.04	≤0.001				****	****	****
Ventral attention	0.46±0.14	0.48±0.16	0.46±0.14	0.41±0.14	0.42±0.15	0.41±0.17	0.33±0.14	4.09	0.02	≤0.001				*	**	****
Dorsal attention B	0.48±0.17	0.52±0.18	0.48±0.15	0.45±0.18	0.45±0.16	0.48±0.21	0.35±0.15	2.81	0.02	≤0.01						****
Dorsal attention A	0.68±0.23	0.72±0.23	0.73±0.20	0.64±0.17	0.64±0.26	0.68±0.26	0.46±0.23	4.15	0.02	≤0.001						****
Somatomotor B	0.67±0.17	0.68±0.20	0.64±0.15	0.59±0.17	0.68±0.18	0.59±0.16	0.55±0.14	4.95	0.03	≤0.001					****	****
Somatomotor A	0.61±0.25	0.64±0.27	0.53±0.21	0.44±0.25	0.50±0.25	0.45±0.25	0.32±0.25	10.94	0.06	≤0.001			***	****	****	****
Visual peripheral	1.05±0.25	1.10±0.30	1.07±0.20	0.99±0.25	0.99±0.24	1.03±0.24	0.89±0.21	1.77	0.01	0.10				*		*
Visual central	0.72±0.31	0.76±0.34	0.83±0.29	0.87±0.26	0.85±0.21	0.84±0.32	0.81±0.21	5.65	0.03	≤0.001		*	*	****	****	*

*Note: Values reflect mean of raw z-transformed Pearson correlation values ± standard deviation across all regional interactions for that network. NTSD: community non-treatment seeking depression; TSD: unipolar treatment seeking depression; BD w/o: bipolar disorder without psychosis; BD w/: bipolar disorder with psychosis; SZ gp1: schizophrenia group 1; SZ gp2: schizophrenia group 2. *P*-values for group comparisons * ≤0.05 **≤0.01 *** ≤0.005 **** ≤0.001

Supplemental Table 3 Cont. Network interactions affected by psychiatric illnesses with or without psychosis

	Non-Treatment Seeking Depression vs					Treatment Seeking Depression vs				Bipolar Disorder (w/o psychosis) vs			Bipolar Disorder (psychosis) vs		Scz Group1 vs Scz Group2
	TSD	Bw/o	B	S1	S2	Bw/o	B	S1	S2	B	S1	S2	S1	S2	
Network	p-values					p-values				p-values			p-values		p-values
Default D	*		****		***								****		*
Default C		***	****	**	***		*								
Default B			****	*	****		***	*	****				*		*
Default A			**		****				*						*
Control C		*	***	****	*										
Control B	*	****	****	****	****		****	*	****				*		*
Control A	**	****	****	****	****				****					*	***
Limbic	*		*		*										
Salience			****	*	*		****	*	*						
Ventral attention					**		*	*	*						
Dorsal attention B			*		****				**						***
Dorsal attention A					****				****			*		*	****
Somatomotor B				*	*								****	****	
Somatomotor A		*		****	****			*	****					***	*
Visual peripheral			*		*		*		*						
Visual central			*	*											

*Note: CD: community depression; UD: unipolar depression; Bw/o: bipolar depression without psychosis; B: bipolar depression with psychosis; S1: schizophrenia group 1; S2: schizophrenia group 2. P-values for group comparisons * ≤0.05 **≤0.01 *** ≤0.005 **** ≤0.001
